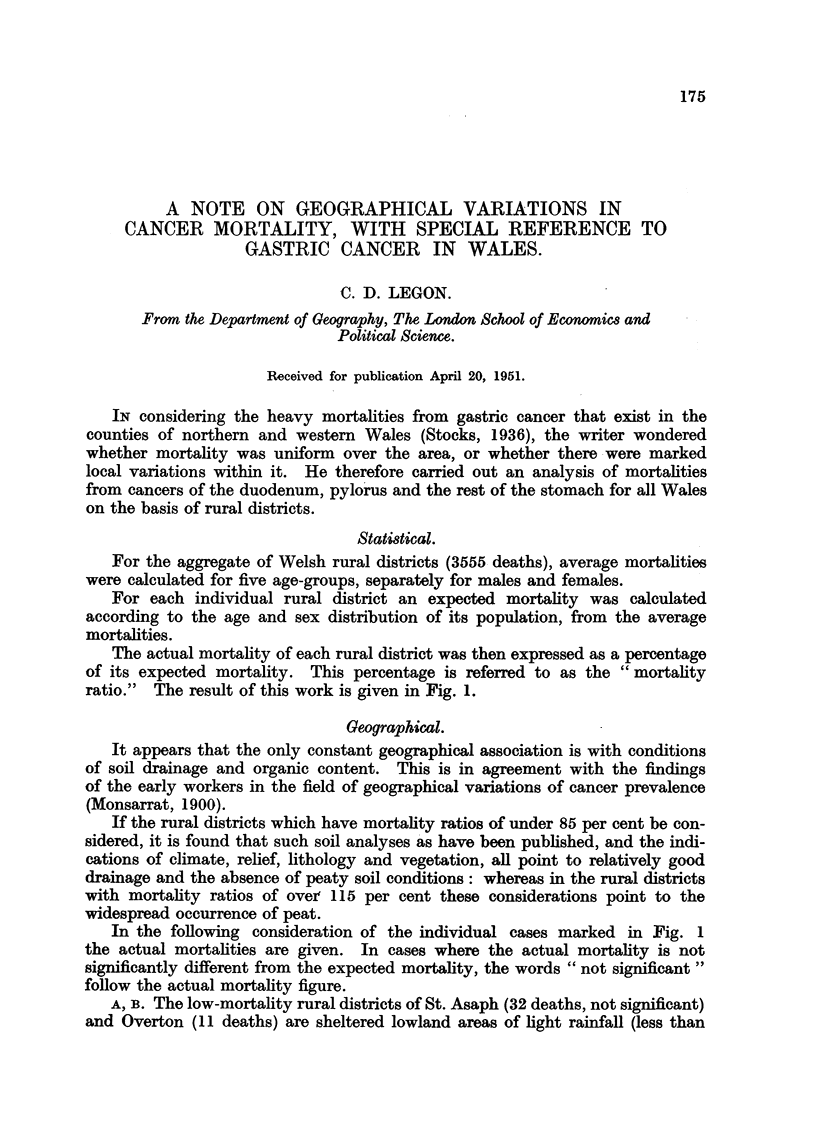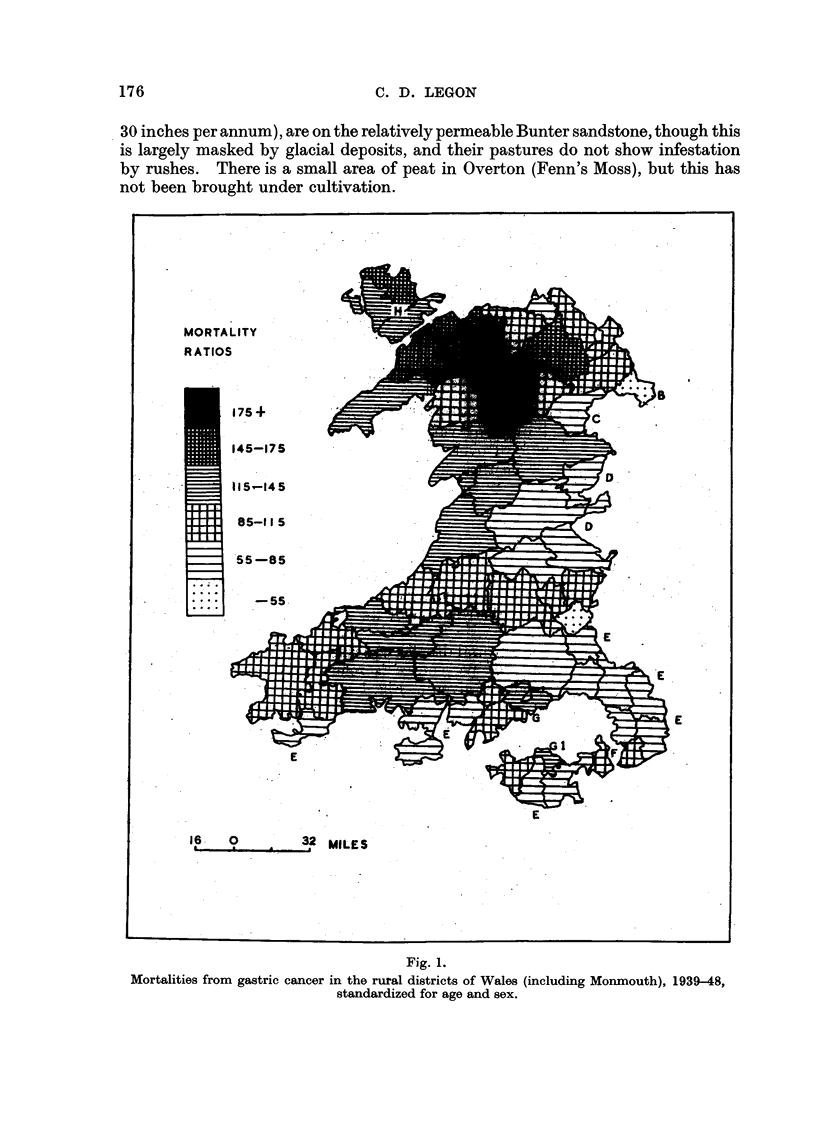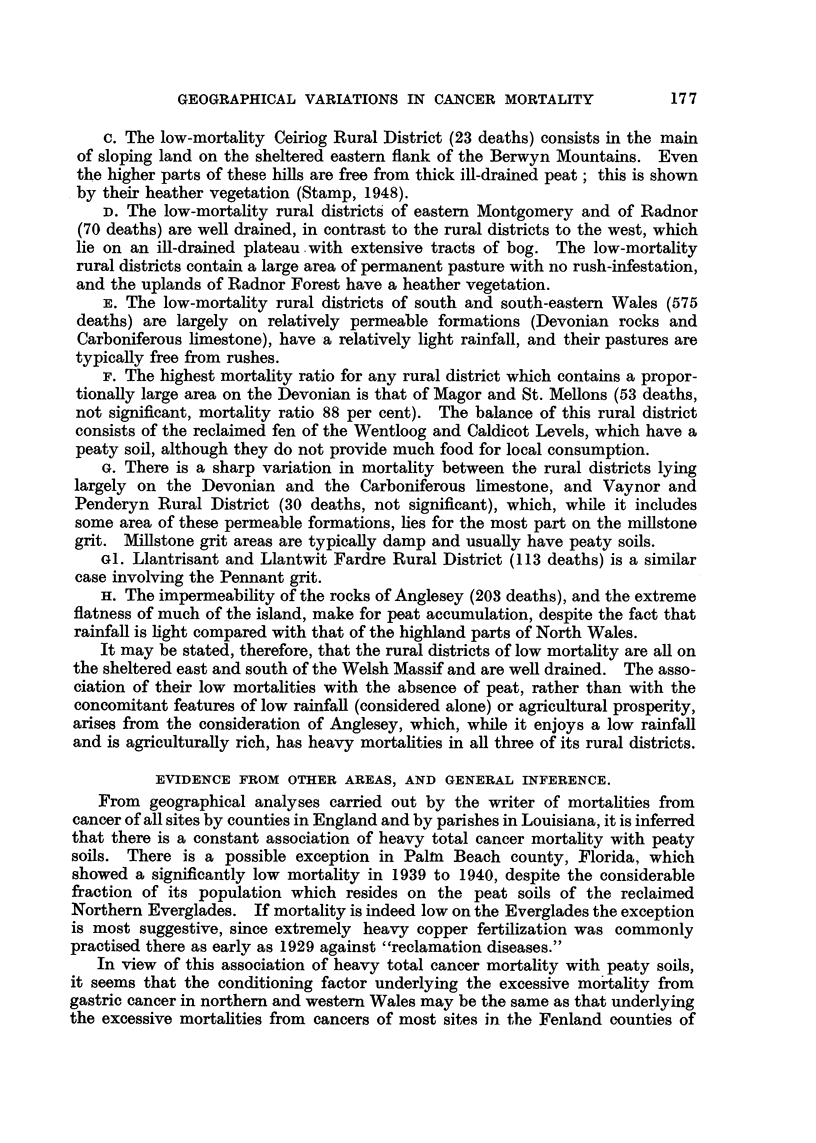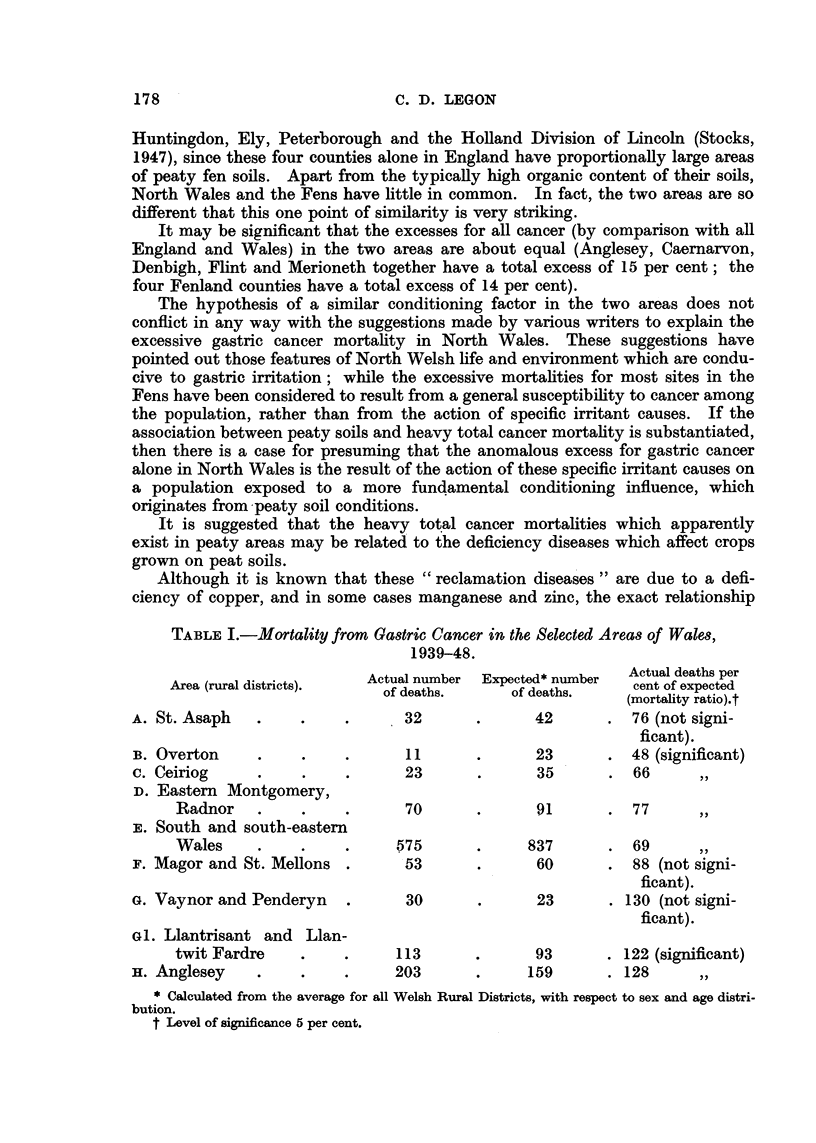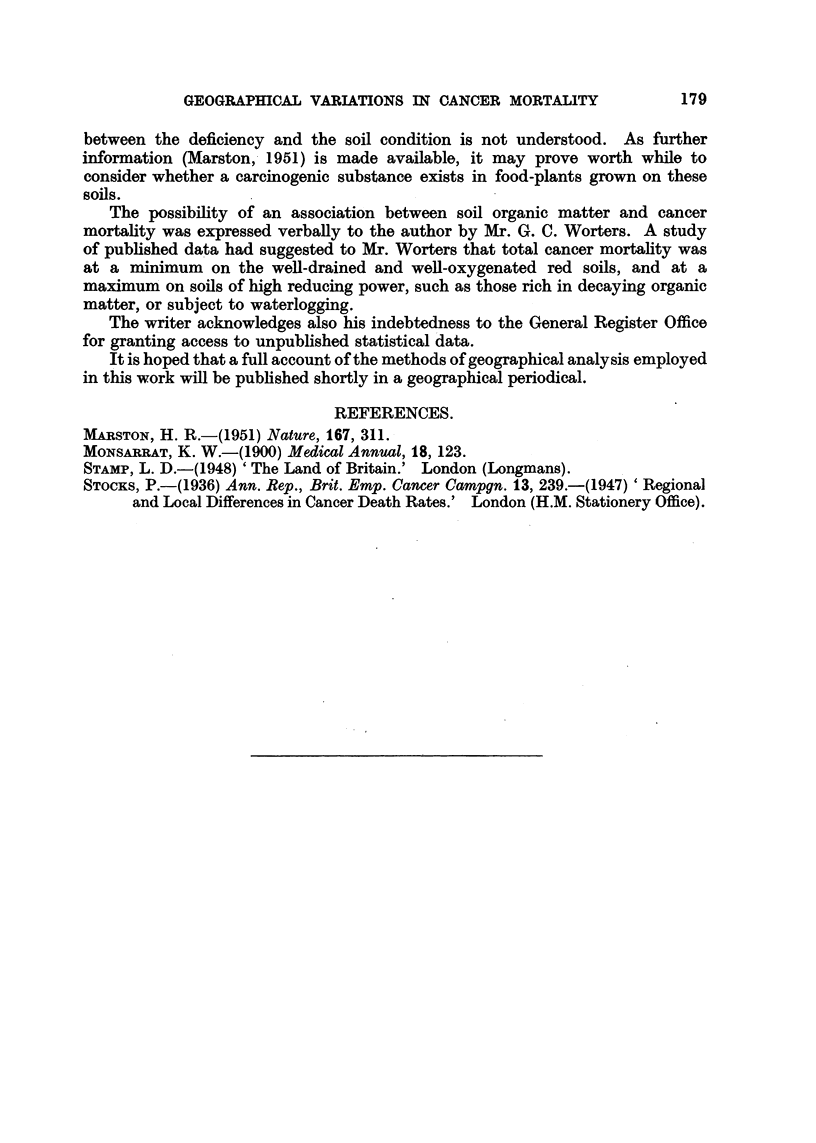# A Note on Geographical Variations in Cancer Mortality, with Special Reference to Gastric Cancer in Wales

**DOI:** 10.1038/bjc.1951.19

**Published:** 1951-06

**Authors:** C. D. Legon


### A NOTE ON GEOGRAPHICAL VARIATIONS IN CANCER MORTALITY, WITH SPECIAL REFERENCE TO GASTRIC CANCER IN WALES.

C. D. LEGON.

*From the Department of Geography, The London School of Economics and Political Science.*

Received for publication April 20, 1951.

IN considering the heavy mortalities from gastric cancer that exist in the counties of northern and western Wales (Stocks, 1936), the writer wondered whether mortality was uniform over the area, or whether there were marked local variations within it. He therefore carried out an analysis of mortalities from cancers of the duodenum, pylorus and the rest of the stomach for all Wales on the basis of rural districts.

*Statistical.*

For the aggregate of Welsh rural districts (3555 deaths), average mortalities were calculated for five age-groups, separately for males and females.

For each individual rural district an expected mortality was calculated according to the age and sex distribution of its population, from the average mortalities.

The actual mortality of each rural district was then expressed as a percentage of its expected mortality. This percentage is referred to as the "mortality ratio." The result of this work is given in Fig. 1.

*Geographical.*

It appears that the only constant geographical association is with conditions of soil drainage and organic content. This is in agreement with the findings of the early workers in the field of geographical variations of cancer prevalence (Monsarrat, 1900).

If the rural districts which have mortality ratios of under 85 per cent be considered, it is found that such soil analyses as have been published, and the indications of climate, relief, lithology and vegetation, all point to relatively good drainage and the absence of peaty soil conditions : whereas in the rural districts with mortality ratios of over 115 per cent these considerations point to the widespread occurrence of peat.

In the following consideration of the individual cases marked in Fig. 1 the actual mortalities are given. In cases where the actual mortality is not significantly different from the expected mortality, the words "not significant" follow the actual mortality figure.

A, B. The low-mortality rural districts of St. Asaph (32 deaths, not significant) and Overton (11 deaths) are sheltered lowland areas of light rainfall (less than

30 inches per annum), are on the relatively permeable Bunter sandstone, though this is largely masked by glacial deposits, and their pastures do not show infestation by rushes. There is a small area of peat in Overton (Fenn's Moss), but this has not been brought under cultivation.

Fig. 1.
Mortalities from gastric cancer in the rural districts of Wales (including Monmouth), 1939–48, standardized for age and sex.

C. The low-mortality Ceiriog Rural District (23 deaths) consists in the main of sloping land on the sheltered eastern flank of the Berwyn Mountains. Even the higher parts of these hills are free from thick ill-drained peat ; this is shown by their heather vegetation (Stamp, 1948).

D. The low-mortality rural districts of eastern Montgomery and of Radnor (70 deaths) are well drained, in contrast to the rural districts to the west, which lie on an ill-drained plateau with extensive tracts of bog. The low-mortality rural districts contain a large area of permanent pasture with no rush-infestation, and the uplands of Radnor Forest have a heather vegetation.

E. The low-mortality rural districts of south and south-eastern Wales (575 deaths) are largely on relatively permeable formations (Devonian rocks and Carboniferous limestone), have a relatively light rainfall, and their pastures are typically free from rushes.

F. The highest mortality ratio for any rural district which contains a proportionally large area on the Devonian is that of Magor and St. Mellons (53 deaths, not significant, mortality ratio 88 per cent). The balance of this rural district consists of the reclaimed fen of the Wentloog and Caldicot Levels, which have a peaty soil, although they do not provide much food for local consumption.

G. There is a sharp variation in mortality between the rural districts lying largely on the Devonian and the Carboniferous limestone, and Vaynor and Penderyn Rural District (30 deaths, not significant), which, while it includes some area of these permeable formations, lies for the most part on the millstone grit. Millstone grit areas are typically damp and usually have peaty soils.

G1. Llantrisant and Llantwit Fardre Rural District (113 deaths) is a similar case involving the Pennant grit.

H. The impermeability of the rocks of Anglesey (203 deaths), and the extreme flatness of much of the island, make for peat accumulation, despite the fact that rainfall is light compared with that of the highland parts of North Wales.

It may be stated, therefore, that the rural districts of low mortality are all on the sheltered east and south of the Welsh Massif and are well drained. The association of their low mortalities with the absence of peat, rather than with the concomitant features of low rainfall (considered alone) or agricultural prosperity, arises from the consideration of Anglesey, which, while it enjoys a low rainfall and is agriculturally rich, has heavy mortalities in all three of its rural districts.

### EVIDENCE FROM OTHER AREAS, AND GENERAL INFERENCE.

From geographical analyses carried out by the writer of mortalities from cancer of all sites by counties in England and by parishes in Louisiana, it is inferred that there is a constant association of heavy total cancer mortality with peaty soils. There is a possible exception in Palm Beach county, Florida, which showed a significantly low mortality in 1939 to 1940, despite the considerable fraction of its population which resides on the peat soils of the reclaimed Northern Everglades. If mortality is indeed low on the Everglades the exception is most suggestive, since extremely heavy copper fertilization was commonly practised there as early as 1929 against "reclamation diseases."

In view of this association of heavy total cancer mortality with peaty soils, it seems that the conditioning factor underlying the excessive mortality from gastric cancer in northern and western Wales may be the same as that underlying the excessive mortalities from cancers of most sites in the Fenland counties of

Huntingdon, Ely, Peterborough and the Holland Division of Lincoln (Stocks, 1947), since these four counties alone in England have proportionally large areas of peaty fen soils. Apart from the typically high organic content of their soils, North Wales and the Fens have little in common. In fact, the two areas are so different that this one point of similarity is very striking.

It may be significant that the excesses for all cancer (by comparison with all England and Wales) in the two areas are about equal (Anglesey, Caernarvon, Denbigh, Flint and Merioneth together have a total excess of 15 per cent; the four Fenland counties have a total excess of 14 per cent).

The hypothesis of a similar conditioning factor in the two areas does not conflict in any way with the suggestions made by various writers to explain the excessive gastric cancer mortality in North Wales. These suggestions have pointed out those features of North Welsh life and environment which are conducive to gastric irritation; while the excessive mortalities for most sites in the Fens have been considered to result from a general susceptibility to cancer among the population, rather than from the action of specific irritant causes. If the association between peaty soils and heavy total cancer mortality is substantiated, then there is a case for presuming that the anomalous excess for gastric cancer alone in North Wales is the result of the action of these specific irritant causes on a population exposed to a more fundamental conditioning influence, which originates from peaty soil conditions.

It is suggested that the heavy total cancer mortalities which apparently exist in peaty areas may be related to the deficiency diseases which affect crops grown on peat soils.

Although it is known that these "reclamation diseases" are due to a deficiency of copper, and in some cases manganese and zinc, the exact relationship

TABLE I.—*Mortality from Gastric Cancer in the Selected Areas of Wales, 1939–48.*

| Area (rural districts). | Actual number of deaths. | Expected* number of deaths. | Actual deaths per cent of expected (mortality ratio).† |
|---|---|---|---|
| A. St. Asaph | 32 | 42 | 76 (not significant). |
| B. Overton | 11 | 23 | 48 (significant) |
| C. Ceiriog | 23 | 35 | 66 ,, |
| D. Eastern Montgomery, Radnor | 70 | 91 | 77 ,, |
| E. South and south-eastern Wales | 575 | 837 | 69 ,, |
| F. Magor and St. Mellons | 53 | 60 | 88 (not significant). |
| G. Vaynor and Penderyn | 30 | 23 | 130 (not significant). |
| G1. Llantrisant and Llantwit Fardre | 113 | 93 | 122 (significant) |
| H. Anglesey | 203 | 159 | 128 ,, |

* Calculated from the average for all Welsh Rural Districts, with respect to sex and age distribution.

† Level of significance 5 per cent.

between the deficiency and the soil condition is not understood. As further information (Marston, 1951) is made available, it may prove worth while to consider whether a carcinogenic substance exists in food-plants grown on these soils.

The possibility of an association between soil organic matter and cancer mortality was expressed verbally to the author by Mr. G. C. Worters. A study of published data had suggested to Mr. Worters that total cancer mortality was at a minimum on the well-drained and well-oxygenated red soils, and at a maximum on soils of high reducing power, such as those rich in decaying organic matter, or subject to waterlogging.

The writer acknowledges also his indebtedness to the General Register Office for granting access to unpublished statistical data.

It is hoped that a full account of the methods of geographical analysis employed in this work will be published shortly in a geographical periodical.

### REFERENCES.

MARSTON, H. R.—(1951) *Nature*, **167**, 311.
MONSARRAT, K. W.—(1900) *Medical Annual*, **18**, 123.
STAMP, L. D.—(1948) 'The Land of Britain.' London (Longmans).
STOCKS, P.—(1936) *Ann. Rep., Brit. Emp. Cancer Campgn.* **13**, 239.—(1947) 'Regional and Local Differences in Cancer Death Rates.' London (H.M. Stationery Office).